# Can nirmatrelvir/ritonavir treatment shorten the duration of COVID-19 isolation?

**DOI:** 10.3389/fmed.2022.988559

**Published:** 2022-10-12

**Authors:** Haein Kim, Jeong-Sun Yang, Jae-Hoon Ko, Myungsun Lee, Joo-Yeon Lee, Sehee Park, Jun-Won Kim, Younmin Shin, Jung-Min Lee, Yoo Jin Na, Byoung Kwon Park, Hyungjin Kim, Young Ho Lee, Jinyoung Yang, Kyungmin Huh, Sun Young Cho, Cheol-In Kang, Doo Ryeon Chung, Kyong Ran Peck

**Affiliations:** ^1^Division of Infectious Diseases, Department of Medicine, Samsung Medical Center, Sungkyunkwan University School of Medicine, Seoul, South Korea; ^2^Center for Emerging Virus Research, Korea National Institute of Health, Korea Disease Control and Prevention Agency, Cheongju-si, South Korea; ^3^Department of Medical Humanities, Samsung Medical Center, Sungkyunkwan University School of Medicine, Seoul, South Korea

**Keywords:** SARS-CoV-2, nirmatrelvir, culture, microbial viability, viral load

## Abstract

**Background:**

The impact of nirmatrelvir/ritonavir treatment on shedding of viable virus in severe acute respiratory syndrome coronavirus 2 (SARS-CoV-2) is unclear.

**Methods:**

A prospective cohort study evaluating mildly ill COVID-19 patients was conducted. Virologic responses were compared between nirmatrelvir/ritonavir-treatment and supportive care groups. Risk factors and relevant clinical factors for shedding of viable virus were investigated.

**Results:**

A total of 80 COVID-19 patients were enrolled and 222 sputum specimens were collected. Ten patients were dropped during follow-up, and 33 patients in the nirmatrelvir/ritonavir and 37 in the supportive care groups were compared. The median age was 67 years, and 67% were male. Clinical characteristics were similar between groups. Viral loads decreased significantly faster in the nirmatrelvir/ritonavir group compared with the supportive care group (*P* < 0.001), and the slope was significantly steeper (–2.99 ± 1.54 vs. –1.44 ± 1.52; *P* < 0.001). The duration of viable virus shedding was not statistically different between groups. In the multivariable analyses evaluating all collected specimens, male gender (OR 2.51, 95% CI 1.25–5.03, *P* = 0.010), symptom score (OR 1.41, 95% CI 1.07–1.87, *P* = 0.015), days from symptom onset (OR 0.72, 95% CI 0.59–0.88, *P* = 0.002), complete vaccination (OR 0.09, 95% CI 0.01–0.87, *P* = 0.038), and BA.2 subtype (OR 0.49, 95% CI 0.26–0.91, *P* = 0.025) were independently associated with viable viral shedding, while nirmatrelvir/ritonavir treatment was not.

**Conclusion:**

Nirmatrelvir/ritonavir treatment effectively reduced viral loads of SARS-CoV-2 Omicron variants but did not decrease the duration of viable virus shedding.

## Summary

In a prospective cohort study, nirmatrelvir/ritonavir treatment effectively reduced viral loads of SARS-CoV-2 Omicron variants but did not decrease the duration of shedding of viable virus. The recommended duration of isolation of COVID-19 patients should be determined regardless of antiviral treatment.

## Introduction

The Omicron variant (B.1.1.529) became the fifth variant of concern (VOC) on November 26, 2021, and dominated the global severe acute respiratory syndrome coronavirus 2 (SARS-CoV-2) outbreak during the first half of 2022 (1). Due to the unprecedented rapid transmission and large outbreak surge of the Omicron variant, healthcare authorities in several countries shortened the isolation period of mildly ill coronavirus disease 2019 (COVID-19) patients to 5 days from symptom onset in order to maintain business continuity and daily life (2, 3). This decision was based on the report that most SARS-CoV-2 transmission occurs within 3 days of symptom onset, in addition to an increase in the proportion of vaccinated individuals in the populations of many countries (2–4). However, it was still recommended to wear a well-fitting mask and take additional precautions for an additional 5 days since viral culture and modeling studies suggested that an infected individual would shed viable virus for 10 days from symptom onset and transmission may occur during this period (2, 5–9).

To date, three antiviral agents against SARS-CoV-2, remdesivir (Veklury; Gilead Sciences, California, USA), nirmatrelvir/ritonavir (Paxlovid; Pfizer, New York, USA), and molnupiravir (Lagevrio; MSD, New Jersey, USA) have received approval or emergency use authorizations (EUA) from the US Food and Drug Administration (FDA) (10). Among these agents, nirmatrelvir/ritonavir is the only highly effective oral antiviral agent, reducing the risk of progression from milder infection to severe COVID-19 by 89% (10–13). It was also reported that nirmatrelvir/ritonavir treatment was associated with a significant reduction in SARS-CoV-2 viral load compared to placebo, but there have been no investigations addressing whether shedding of viable virus is also reduced by nirmatrelvir/ritonavir and if the length of the isolation period of treated patients can consequently be reduced (13). To answer the question, we conducted a prospective cohort study evaluating viable SARS-CoV-2 shedding among nirmatrelvir/ritonavir-treated and non-treated mildly ill COVID-19 patients.

## Materials and methods

### Study population and design

A prospective observational cohort study was conducted to evaluate the effectiveness of nirmatrelvir/ritonavir on virologic outcomes of mildly ill COVID-19 patients who were managed at a residential care center in Seoul, South Korea. The residential care center was temporarily operated between October 2021 and May 2022, and mildly ill COVID-19 patients at risk of disease progression were managed at the center. Patients with the following inclusion criteria, adopted from the nirmatrelvir/ritonavir treatment indications, were screened between January 2022 and March 2022: (1) Confirmed SARS-CoV-2 infection, (2) symptom onset within 4 days before admission, (3) no oxygen requirement (SpO_2_ > 94% in room air), and (4) high risk for disease progression (age ≥ 40 years plus one of the underlying diseases including diabetes mellitus, cardiovascular disease, chronic renal disease, chronic lung disease, and body mass index (BMI) ≥ 30 kg/m^2^, or age ≥ 60 years with or without underlying disease). Confirmation of SARS-CoV-2 infection was performed using a real-time reverse transcription polymerase chain reaction (RT-PCR) assay test kit approved by the Korean Ministry of Food and Drug Safety (14). Patients with severe comorbidities requiring hospital care were excluded.

The screened patients were notified about the benefits and potential side effects of nirmatrelvir/ritonavir treatment and made their own decision about whether to take the drug. After a detailed explanation of the study, patients who provided verbal consent were enrolled in the study with a target of forty patients for each of the nirmatrelvir/ritonavir and supportive care groups. In the nirmatrelvir/ritonavir group, 300 mg nirmatrelvir with 100 mg ritonavir was administered orally every 12 h for 5 days (10 doses total), beginning the day after admission (Day 1), because some patients were admitted to the center during the evening time (Day 0). Oral medications for symptom control, including acetaminophen, antihistamines, pseudoephedrine, and antitussive agents, were provided for patients in both groups as ordered by the attending physicians. Each patient provided a 2 mL volume of early morning sputum on Day 1 (baseline), Day 3, and Day 5 (discharge day). Review by the Institutional Review Board (IRB) was not required because the present investigation was conducted as a part of a public health response and minimal risk was expected to the participating patients (IRB of Samsung Medical Center, IRB review exemption number:2022-01-179).

### Data collection

Information about baseline characteristics including age, gender, date of symptom onset/diagnosis/admission, underlying diseases, initial vital signs, initial chest X-ray findings, and COVID-19 vaccination (BNT162b2, mRNA-1273, and ChAdOx1) doses were collected. Information about COVID-19-related symptoms was collected by questionnaires once daily (8 AM) for 5 days using a telephone interview administered by the medical staff. A total of 14 COVID-19-related symptoms were categorized within seven groups (fever/chill/myalgia, rhinorrhea/nasal stuffiness/congestion, cough/sputum, sore throat, chest pain/discomfort, headache, and nausea/diarrhea/abdomen discomfort). Each symptom group was scored as 1 point and evaluated every day from Day 0 (admission day) to Day 5 (discharge day).

### Laboratory procedures

Detection and quantification of SARS-CoV-2 viral load in sputum specimens were assessed using PowerCheck™ SARS-CoV-2 RT-PCR kits (KogenBiotech, Seoul, Korea), and identification of SARS-CoV-2 variants was performed using the PowerCheck™ SARS-CoV-2 S-gene Mutation Detection Kit Ver.3.0 (KogenBiotech). We converted the resulting cycle threshold (Ct) values to log_10_ RNA copies/mL using calibration curves based on quantified ORF1ab *in vitro* RNA transcripts (5). Isolation of infectious SARS-CoV-2 from specimens was performed to define the duration of viable virus shedding. Vero E6 cells were seeded into 24-wall plates at 1.5 × 10^5^ cells/well 24 h prior to virus isolation assays. Diluted samples were inoculated into the cells, incubated for 1 h (37°C, 5% CO_2_) with rocking every 15 min, and 1 mL of Dulbecco’s modified Eagle’s medium supplemented with 2% fetal bovine serum and penicillin-streptomycin was added after removal of the inoculum. Cell morphology was examined under a microscope daily for 7 days post-inoculum to detect cytopathic effects (CPE). A positive viral culture was suspected when SARS-CoV-2-specific CPE were observed in the inoculated cells and confirmed by real-time RT-PCR. Inoculum with positive CPE with high SARS-CoV-2 RNA (> 10^6^ copies/mL) were considered culture-positive, all other results were considered culture-negative. Patients with positive CPE and low SARS-CoV-2 RNA copy numbers were supposed to undergo a repeated test, but no such case was observed during the present experiment. A specimen that exhibited positive culture by the first or second cell passage was defined as containing viable SARS-CoV-2 for the statistical analyses. All procedures were performed in a level-3 biosafety facility according to the laboratory biosafety guidelines of the Korea Disease Control and Prevention Agency.

### Statistical analyses

We analyzed categorical variables using the *chi*-square or Fisher’s exact test and continuous variables using Student’s *t*-test or the Mann–Whitney *U*-test. The changes in symptom scores and viral loads between the two groups over the study period were evaluated using a generalized linear mixed model. Since the initial viral loads of each patient were different, we calculated the changes in viral load individually and compared calculated viral load reductions between groups. Slopes of viral loads were analyzed by linear regression. Factors for viable virus shedding were analyzed for each specimen. Univariable analyses of factors influencing viable viral shedding were performed using a logistic regression model, and variables with *P* < 0.1 were included in multivariable analyses. All tests of significance were two-tailed, and *P* < 0.05 was considered statistically significant. All statistical analyses were performed using SPSS^®^, version 27.0 K for the Windows software package (SPSS Inc., Chicago, IL, USA), and GraphPad Prism 9.0 (GraphPad Software, San Diego, CA, USA) was used to develop the figures.

## Results

### Baseline characteristics of mildly ill COVID-19 patients

**FIGURE 1:**
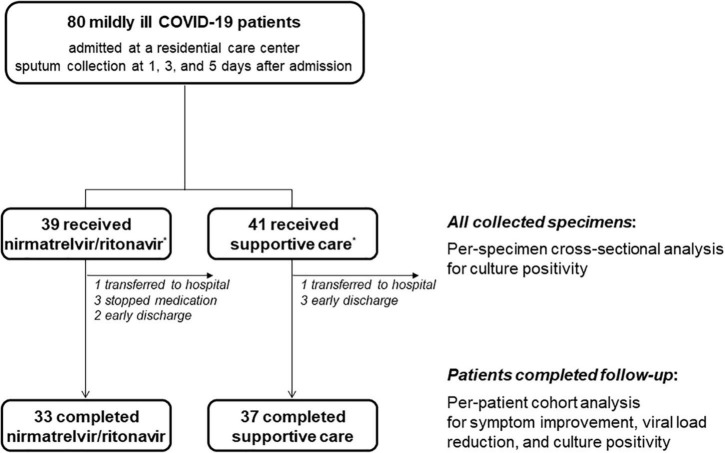
Schematic flow of patient enrollment and specimen collection. *The target population was 40 patients for each group, but 39 patients who received nirmatrelvir/ritonavir and 41 who underwent supportive care group were finally enrolled due to a labeling error.

During the study period, a total of 80 mildly ill COVID-19 patients were enrolled; 39 received nirmatrelvir/ritonavir and 41 received supportive care (Figure 1). After enrollment, two patients were transferred to a hospital for management of medical conditions (skin rash and diarrhea), three stopped the nirmatrelvir/ritonavir treatment due to adverse effects (diarrhea, elevated blood pressure, and abdominal discomfort), and five were discharged early. All collected specimens were used for per-specimen analyses for culture positivity, and the 70 patients who completed the study were included in the per-patient analyses.

The baseline characteristics of the study population of 70 mildly ill COVID-19 patients are presented in Table 1. The median age was 67 years, and 47 patients (67%) were male. Patients were admitted to the residential care center within a median of 3 days from symptom onset (IQR 2–4 days). The most common comorbidities were hypertension (51%), followed by dyslipidemia (40%) and diabetes mellitus (26%). The most common symptoms were cough/sputum (87%), followed by sore throat (73%) and rhinorrhea/nasal stuffiness/congestion (56%). Symptom scores decreased after admission without a significant difference between the two groups (Supplementary Figure 1). Most patients (86%) received boosting vaccination. There were no significant differences with respect to age, gender, duration of symptoms before admission, underlying diseases, COVID-19-related symptoms, and vaccination doses between the nirmatrelvir/ritonavir and supportive care groups, except among those with chronic lung disease (15.2 and 0%, for the treated and supportive care groups, respectively; *P* = 0.020). All patients were infected with an Omicron variant, and half of them had the BA.2 Omicron variant.

**TABLE 1:** Clinical characteristics of the mildly ill COVID-19 study patients.

Variables	Nirmatrelvir/ Ritonavir (*n* = 33)	Supportive care (*n* = 37)	*P*-value
**Age, years**	67 (61–74)	67 (60–73)	0.469
**Male gender**	19 (57.6)	28 (75.7)	0.108
**Interval from symptom onset to admission**	3 (2–4)	3 (1–4)	0.119
**Underlying disease**			
Hypertension	15 (45.5)	21 (56.8)	0.345
Diabetes mellitus	6 (18.2)	12 (32.4)	0.173
Dyslipidemia	13 (39.4)	15 (40.5)	0.922
Solid tumor, treated	0	4 (10.8)	0.117
Cardiovascular disease	1 (3.0)	7 (18.9)	0.058
Chronic lung disease	5 (15.2)	0	0.020
Chronic liver disease	0	2 (5.4)	0.494
**Symptom/s at admission**			
Fever/chill/myalgia	19 (57.6)	18 (48.6)	0.455
Rhinorrhea/nasal stuffiness	18 (54.5)	21 (56.8)	0.853
Cough/sputum	30 (90.9)	31 (83.8)	0.485
Sore throat	24 (72.7)	27 (73.0)	0.982
Chest pain/dyspnea	5 (15.2)	9 (24.3)	0.338
Headache	3 (9.1)	2 (5.4)	0.661
Nausea/diarrhea/ abdominal discomfort	6 (18.2)	11 (29.7)	0.261
Symptom scores	3 (3–4)	3 (2–4)	0.434
**SpO_2_ at admission,%**	97 (97–98)	97 (96–98)	0.317
**Temperature at admission, °C**	36.2 (35.7–36.8)	36.0 (35.6–36.5)	0.080
**Vaccination**			
None	2 (6.1)	0	0.219
Completed	3 (9.1)	5 (13.5)	0.714
Boosted	28 (84.8)	32 (86.5)	> 0.999
**SARS-CoV-2 viral load, log**_10_ **copies/mL**	8.34 (7.50–9.11)	8.62 (7.90–9.05)	0.287
**SARS-CoV-2 virus subtype**			
Omicron variant, BA.1	13 (39.4)	22 (59.5)	0.094
Omicron variant, BA.2	20 (60.6)	15 (40.5)	0.094

Data are expressed as the number (%) of patients or median (IQR). COVID-19, coronavirus disease 2019; SARS-CoV-2; severe acute respiratory syndrome coronavirus 2; IQR, interquartile range.

### Viral load according to nirmatrelvir/ritonavir treatment

**FIGURE 2:**
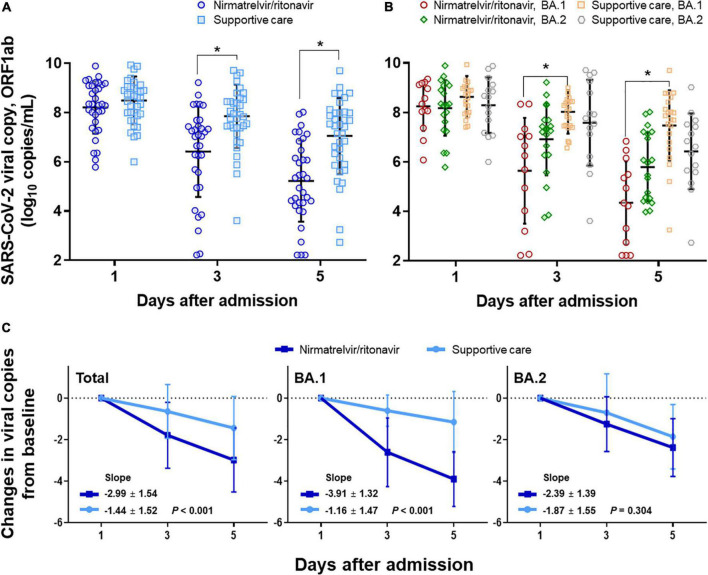
Changes in viral load in response to nirmatrelvir/ritonavir treatment compared with supportive care. **(A)** Changes in viral load in response to nirmatrelvir/ritonavir or supportive care, total patients. **(B)** Subgroup analyses among patients with BA.1 variant infection and BA.2 variant infection, respectively. **(C)** Changes in viral copy numbers after adjusting for baseline viral loads. *Statistically significant.

Changes in viral load were compared in the nirmatrelvir/ritonavir and supportive care groups (Figure 2). The mean viral loads decreased significantly from 8.2 to 5.2 log_10_ copies/mL in the nirmatrelvir/ritonavir group (*P* for time effect < 0.001) and also fell from 8.5 to 7.0 log_10_ copies/mL in the supportive care group (*P* for time effect < 0.001). There was a significant interaction between the groups and the time (*P* for interaction < 0.001; Figure 2A). When the viral loads were compared at each time point, the initial viral loads were similar in the nirmatrelvir/ritonavir and supportive care groups (average 8.3 vs. 8.6 log_10_ copies/mL, respectively, *P* = 0.287). The mean viral loads were significantly lower in the nirmatrelvir/ritonavir group compared with the supportive-care group on Days 3 and 5 (both *P* < 0.001), mainly due to reductions in BA.1 variant infection (Figure 2B). In subgroup analyses, among patients infected with the BA.1 variant, the initial viral loads were similar between the nirmatrelvir/ritonavir and supportive care groups (8.7 vs. 9.0 log_10_ copies/mL, *P* = 0.241). The mean viral loads in patients infected with the BA.1 variant were significantly lower in the nirmatrelvir/ritonavir group compared with the supportive-care group on Days 3 and 5 (*P* = 0.002 and *P* < 0.001, respectively), with significant interaction between the treatment group and time (*P* for interaction < 0.001). There were no statistically significant differences between the two groups with respect to changes in mean viral loads in patients infected with the BA.2 variant on Days 1, 3, and 5 (*P* = 0.770, 0.226, and 0.210, respectively), with no significant interaction between the treatment group and time (*P* for interaction = 0.327). After adjusting for baseline viral load, we evaluated viral load reduction along the timeline. The slope of the plot of the reduction viral load with time was significantly steeper in the nirmatrelvir/ritonavir group (–3.0 ± 1.5) compared with the supportive care group (–1.4 ± 1.5; *P* < 0.001), mainly attributable to patients infected with the BA.1 variant (*P* < 0.001 for BA.1 and *P* = 0.304 for BA.2; Figure 2C).

### Culture-positive virus shedding according to nirmatrelvir/ritonavir treatment

**FIGURE 3:**
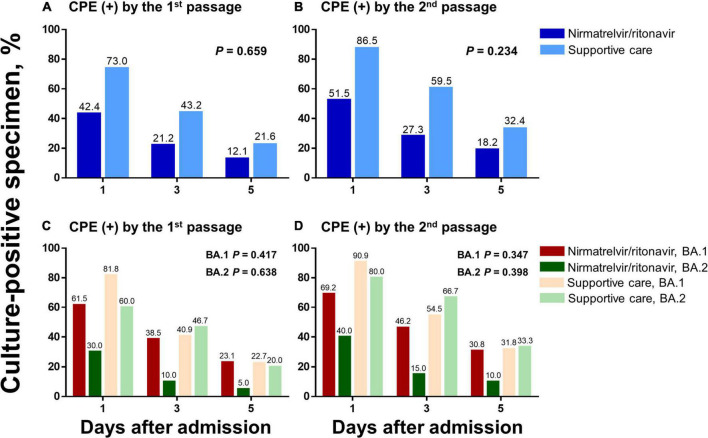
Changes in culture-positive viral shedding in response to nirmatrelvir/ritonavir treatment compared with supportive care. Changes in culture-positive viral shedding are compared in the nirmatrelvir/ritonavir and supportive care groups, among the total study population **(A,B)** and according to the viral subtypes **(C,D)**. Two different criteria for positive culture were applied: (1) CPE-positive by the first cell passage (a and c) and (2) CPE-positive by the second cell passage **(B,D)**. CPE, cytopathic effects.

Changes in culture-positive virus shedding were compared between the nirmatrelvir/ritonavir and supportive care groups (Figure 3). When the percentages of patients with positive CPE by the first cell passage were compared, 42.4% of patients were culture-positive in the nirmatrelvir/ritonavir group on Day 1 and 12.1% on Day 5 (*P* for trend = 0.005), while culture-positive patients decreased from 73.0 to 21.6% in the supportive care group (*P* for trend < 0.001). There were no significant interactions between the two groups and the time (*P* for interaction = 0.659). When the percentages of patients with positive CPE by the second cell passage (definition of viable virus used in the present study) were compared, 51.5% of patients in the nirmatrelvir/ritonavir group were culture-positive on Day 1 and 18.2% on Day 5 (*P* for trend = 0.004), while the percentage decreased from 86.5 to 32.4%, respectively, in the supportive care group (*P* for trend < 0.001). There were no statistically significant differences between the two groups (*P* for interaction = 0.234). In the subgroup analyses comparing patients infected with the BA.1 and BA.2 variants, similar trends were noted.

### Per-specimen analyses to identify risk factors for shedding of viable virus

**FIGURE 4:**
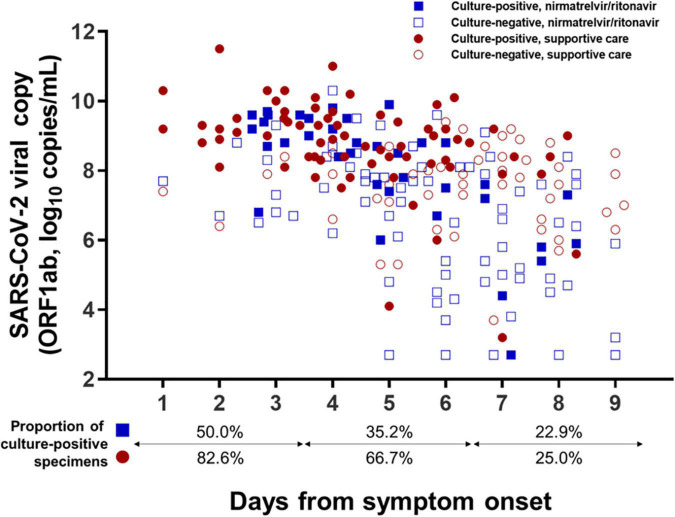
Distribution of specimens with viable virus according to days from symptom onset. Culture results from a total of 222 sputum specimens obtained from 80 mildly ill COVID-19 patients are presented according to the number of days from symptom onset. Specimens exhibiting positive viral culture by the second cell passage were defined as containing viable SARS-CoV-2.

A total of 222 sputum specimens obtained from 80 mildly ill COVID-19 patients were submitted for viral culture: 107 were obtained from the nirmatrelvir/ritonavir group and 115 from the supportive care group, including specimens obtained from patients who did not complete follow-up. For the per-specimen analyses, viable virus shedding was defined as CPE-positive cultures by the second cell passage. The distributions of culture-positive sputum specimens according to the number of days from symptom onset and viral loads are shown in Figure 4. Shedding of viable virus was detectable up to 8 days from symptom onset in both groups, while all the specimens collected at 9 days from symptom onset were culture-negative. When the timelines were grouped with a 3-day interval, the percentage of virus-positive cultures decreased significantly from 50.0% of specimens collected on Days 1–3 to 22.9% of those collected on Days 7–9 in the nirmatrelvir/ritonavir group (*P* = 0.032 for trend), and also fell from 82.6 to 25.0% in the supportive-care group (*P* < 0.001 for trend).

To identify risk factors for the shedding of viable virus, clinical variables relevant to culture positivity for each specimen were analyzed using a logistic regression model (Table 2). In univariable analyses, male gender, days from symptom onset, symptom score, vaccination doses, nirmatrelvir treatment, and BA.1 subtype were found to have *P*-values of less than 0.1 and were included in the multivariable analyses. The independent risk factors associated with viable viral shedding were found to be male gender (OR 2.51, 95% CI 1.25–5.03, *P* = 0.010) and symptom score (OR 1.41, 95% CI 1.07–1.87, *P* = 0.015), while days from symptom onset (OR 0.72, 95% CI 0.59–0.88, *P* = 0.002), complete vaccination (OR 0.09, 95% CI 0.01–0.87, *P* = 0.038), and BA.2 subtype (OR 0.49, 95% CI 0.26–0.91, *P* = 0.025) were associated with reduced risk for viable virus shedding. The effect of nirmatrelvir/ritonavir treatment was inconsistent: four-dose treatment (specimen collected after 2 days’ treatment) was statistically significant (OR 0.39, 95% CI 0.16–0.95, *P* = 0.038), while eight-dose treatment (specimen collected after 4 days’ treatment) was not (OR 0.48, 95% CI 0.15–1.53, *P* = 0.212).

**TABLE 2:** Univariable and multivariable analyses of all collected specimens to identify factors influencing culture positivity in mildly ill COVID-19 patients.

Variable	Univariable analyses		Multivariable analyses	
	OR (95% CI)	*P*-value	OR (95% CI)	*P*-value
**Age, years**	0.99 (0.96–1.02)	0.458		
**Male gender**	1.94 (1.10–3.42)	0.022	2.51 (1.25–5.03)	0.010
**Days from symptom onset**	0.67 (0.57–0.78)	< 0.001	0.72 (0.59–0.88)	0.002
**Number of underlying diseases**	1.27 (0.96–1.68)	0.097		
**Symptom score**	1.72 (1.35–2.19)	< 0.001	1.41 (1.07–1.87)	0.015
Vaccination doses*				
Incomplete	1			
Complete	0.14 (0.02–1.16)	0.068	0.09 (0.01–0.87)	0.038
**Nirmatrelvir/ritonavir treatment**				
None	1			
4 Doses	0.29 (0.13–0.65)	0.002	0.39 (0.16–0.95)	0.038
8 Doses	0.17 (0.07–0.43)	< 0.001	0.48 (0.15–1.53)	0.212
**SARS-CoV-2 virus subtype**				
BA.1	1			
BA.2	0.52 (0.30–0.88)	0.015	0.49 (0.26–0.91)	0.025

*Incomplete vaccination means none or single dose of vaccination, and complete vaccination means two or three doses of vaccination. COVID-19, coronavirus disease 2019; OR, odd ratio; CI, confidence interval; SARS-CoV-2; severe acute respiratory syndrome coronavirus 2.

## Discussion

In December 2021, the US Food and Drug Administration issued an emergency use authorization for nirmatrelvir/ritonavir to treat mildly to moderately ill COVID-19 patients at high risk for progression to severe illness. A randomized controlled trial assessing the efficacy of nirmatrelvir/ritonavir showed a significant reduction of viral load, but the trial was conducted during a period when the SARS-CoV-2 Delta variant (B.1.617.2) dominated infections and excluded patients who had prior COVID-19 vaccination (13). In addition, the viability of SARS-CoV-2 shed by nirmatrelvir/ritonavir-treated patients has not been evaluated. Although previous studies have assessed the length of the infectious period of COVID-19, it is essential to obtain updated data that reflect changing outbreak situations, including the emergence of new VOC, vaccine uptake in the population, and introduction of oral antiviral agents, to derive appropriate guidelines for the duration of isolation. Therefore, we conducted the present prospective cohort study to evaluate the effect of nirmatrelvir/ritonavir treatment on shedding of viable virus in an Omicron-dominant, widely vaccinated area.

Of note, we found that nirmatrelvir/ritonavir treatment was not associated with more rapid clearance of culture-positive virus on either per-patient or per-specimen analyses. In the comparison between nirmatrelvir/ritonavir and supportive care groups (per-patient analysis), viral load was significantly reduced in the nirmatrelvir/ritonavir treatment group compared with the supportive care group, as noted in previous analyses (13), but there were no differences between the groups with respect to changes in culture-positivity of specimens regardless of definition of positive culture (first or second passage of the cultures) and virus subtype. To adjust for relevant clinical variables that might have influenced culture positivity, we also conducted per-specimen analyses using a logistic regression model. In univariable analyses, nirmatrelvir/ritonavir treatment was significantly associated with a negative effect on culture positivity, which was stronger after eight-dose treatment (OR 0.17, 95% CI 0.07–0.43, *P* < 0.001) than four-dose treatment (OR 0.29, 95% CI 0.13–0.65, *P* = 0.002). However, this association was reversed after adjustment for clinical variables: four-dose treatment was still statistically significant (OR 0.39, 95% CI 0.16–0.95, *P* = 0.038), while eight-dose treatment was not (OR 0.48, 95% CI 0.15–1.53, *P* = 0.212). In addition, days from symptom onset (OR 0.72, CI 0.59–0.88, *P* = 0.002) and complete vaccination (OR 0.09, CI 0.01–0.87, *P* = 0.038) were still significantly associated with a negative effect on viable virus shedding. Considering that positive cultures were identified in specimens collected by 8 days from symptom onset and the eight-dose specimens were collected 2 days later than the four-dose specimens, the effects adjusted by days from symptom onset and the protective immunity of complete vaccination could influence viable virus shedding greater than nirmatrelvir/ritonavir treatment itself. Previous reports also suggested that clearance of viable virus might be most strongly associated with the production of neutralizing antibody, which might become maximal around 1 week after symptom onset (15). Although it is evident that nirmatrelvir/ritonavir treatment reduced the risk of disease progression and viral load, the recommended duration of isolation should be based on the timeline from symptom onset and vaccination status, rather than antiviral treatment.

Currently, the standard method to evaluate viral clearance is based on examining CPE induced by viruses added to cultured cells (16). Previous studies have shown that the median interval from symptom onset to viral clearance in hospitalized patients was 7 days (17), and viral replication occurred for up to 10 days in mildly ill COVID-19 patients, compared with severely ill COVID-19 patients, in whom the virus could remain viable for up to 32 days after symptom onset (18). However, in the present study, viable virus was detected up to 8 days after symptom onset, and no specimen collected 9 days after symptom onset was culture-positive. The interpretation of this finding is limited by the lack of specimens collected more than 9 days from symptom onset because we did not collect specimens after discharge from the residential care center; however, our findings suggested that vaccination might shorten the duration of viable virus shedding. The findings of the present study that the duration of symptoms and full vaccination were strongly associated with viral culture negativity are consistent with previous reports that viral clearance was faster in vaccinated individuals than in the unvaccinated, and that upon seroconversion, infectious virus shedding dropped rapidly and became undetectable over time (15, 19). The period of viable virus shedding may be further reduced when people who have strengthened their immunity through breakthrough infections during the current Omicron outbreak experience re-infection in the future as a result of newly emerging variants. To establish optimal and timely guidelines for the duration of isolation, follow-up studies are needed.

Interestingly, the rapid reduction of viral load in the nirmatrelvir/ritonavir group than in the supportive care group was statistically significant in patients infected with the BA.1 variant (*P* < 0.001), but not in BA.2 variant infections (*P* = 0.304). There are several possible explanations for these differences. The first is that SARS-CoV-2 virus subtypes were unequally distributed in the two groups, although the effect of this distribution was not statistically significant in the subgroup analyses. Second, the protective effect of pre-formed immunity elicited by COVID-19 vaccination may have been less effective when challenged with the BA.2 variant (20, 21). Third, the BA.2 variant that circulated in South Korea may have harbored mechanisms of resistance against nirmatrelvir/ritonavir, an effect that may need further investigation.

This study had several limitations. Firstly, it was conducted in a single residential care center and included a relatively small number of patients with a high risk of progression to severe COVID-19. A large longitudinal multicenter study is needed to increase the statistical power. Secondly, we only collected the specimens up to 5 days from the admission day. During this period, mildly ill COVID-19 patients were managed at the residential care center immediately after the diagnosis of SARS-CoV-2 infection and were discharged 5 days after the initial diagnosis. For a precise evaluation of the duration of viral shedding, collection of specimens is required in each patient until the end of viable virus shedding. Thirdly, we did not collect serum samples due to the limited medical resources of the residential care center and could not investigate associations between neutralizing antibody levels and virus viability. Finally, we did not collect the data about the types of vaccinations received by the patients and could not compare the viral load reduction according to the type of vaccination. However, the main purpose of this study was to evaluate the effect of nirmatrelvir/ritonavir treatment on viable SARS-CoV-2 shedding, rather than evaluating the effectiveness of protective immunity of each vaccination type.

## Conclusion

In conclusion, in a prospective cohort study conducted in a widely vaccinated area, nirmatrelvir/ritonavir treatment effectively reduced viral loads of SARS-CoV-2 Omicron variants but did not reduce the duration of viable virus shedding. The findings indicate the duration of isolation for mildly ill COVID-19 patients should be determined based on the timelines from symptom onset, rather than antiviral treatment.

## Data availability statement

The original contributions presented in this study are included in the article/Supplementary material, further inquiries can be directed to the corresponding authors.

## Ethics statement

The studies involving human participants were reviewed and approved by the Institutional Review Board (IRB) of Samsung Medical Center. The patients/participants provided their written informed consent to participate in this study.

## Author contributions

HaK, J-SY, J-HK, and ML contributed to the conceptualization and writing—review and editing. HaK, J-HK, HyK, YL, and JY contributed to the investigation. J-SY, ML, J-YL, SP, J-WK, YS, J-ML, YN, and BP contributed to the laboratory work and methodology. HaK and J-HK contributed to the statistical analysis. KH, SC, C-IK, DC, and KP contributed to the supervision. All authors contributed to the article and approved the submitted version.
