# Supplementary material for: Can nirmatrelvir/ritonavir treatment shorten the duration of COVID-19 isolation?
Source: Front Med (Lausanne). 2022 Oct 12;9:988559. doi: 10.3389/fmed.2022.988559 (PMC9596971; doi:10.3389/fmed.2022.988559)
Supplement: Supplementary file 1 [file Image_1.pdf]

Supplementary materials

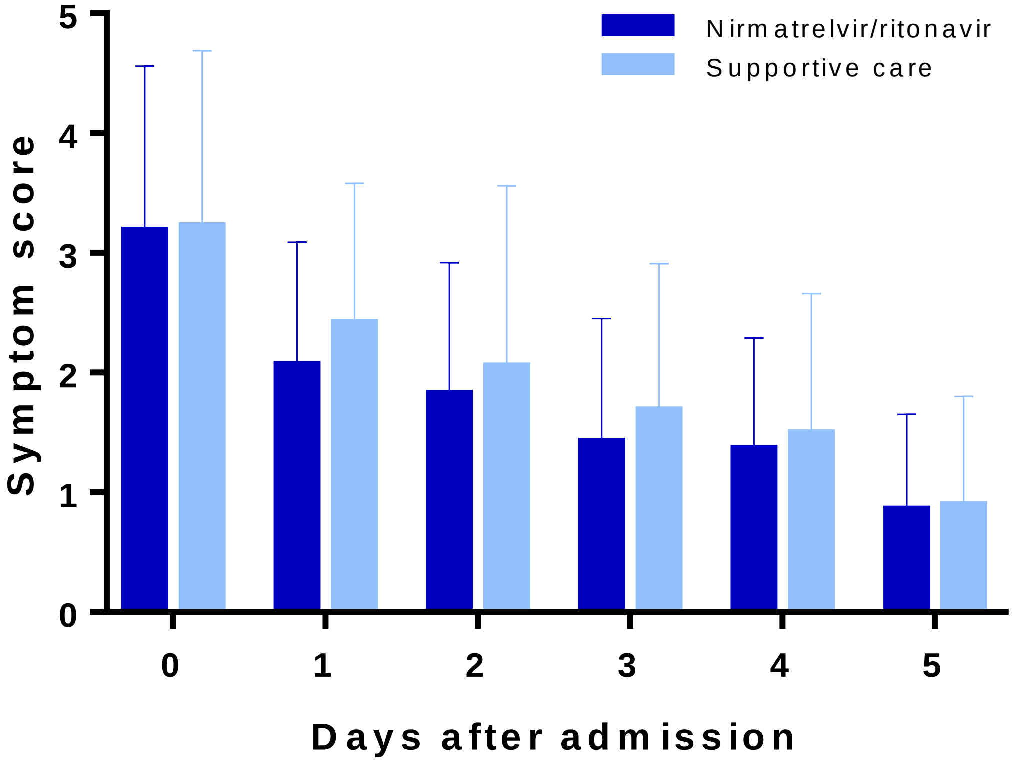

Supplementary Figure 1. Changes in symptom scores in the nirmatrelvir/ritonavir and supportive care groups

The median value of symptom scores decreased significantly from 3.2 to 0.8 in patients in the nirmatrelvir/ritonavir group (*P* for time effect < 0.001), and also fell from 3.2 to 0.9 in patients in the supportive care group (*P* for time effect < 0.001). However, there were no significant differences in the median value of symptom scores between the two groups (*P* for group effect = 0.52) and no significant interaction between the groups and time since admission (*P* for interaction = 0.87).
